# Wnt antagonist, secreted frizzled-related protein 1, is involved in prenatal skeletal muscle development and is a target of miRNA-1/206 in pigs

**DOI:** 10.1186/s12867-015-0035-7

**Published:** 2015-03-08

**Authors:** Yalan Yang, Wei Sun, Ruiqi Wang, Chuzhao Lei, Rong Zhou, Zhonglin Tang, Kui Li

**Affiliations:** Key Laboratory of Farm Animal Genetic Resources and Germplasm Innovation of Ministry of Agriculture, Institute of Animal Science, Chinese Academy of Agricultural Sciences, Beijing, 100193 P.R. China; Agricultural Genome Institute at Shenzhen, Chinese Academy of Agricultural Sciences, Shenzhen, 518124 P.R. China; College of Animal Science and Technology, Northwest A & F University, No. 22 Xinong Road, 712100 Yangling, Shanxi P.R. China

**Keywords:** *SFRP1*, miRNA-206, miRNA-1, Skeletal muscle, Development, Pig

## Abstract

**Background:**

The Wnt signaling pathway is involved in the control of cell proliferation and differentiation during skeletal muscle development. Secreted frizzled-related proteins (SFRPs), such as *SFRP1*, function as inhibitors of Wnt signaling. MicroRNA-1/206(miRNA-1/206) is specifically expressed in skeletal muscle and play a critical role in myogenesis. The miRNA-mRNA profiles and bioinformatics study suggested that the *SFRP1* gene was potentially regulated by miRNA-1/206 during porcine skeletal muscle development.

**Methods:**

To understand the function of *SFRP1* and miRNA-1/206 in swine myogenesis, we first predicted the targets of miRNA-1/206 with the TargetScan and PicTar programs, and analyzed the molecular characterization of the porcine *SFRP1* gene. We performed a temporal-spatial expression analysis of *SFRP1* mRNA and miRNA-206 in Tongcheng pigs (a Chinese indigenous breed) by quantitative real-time polymerase chain reaction, and conducted the co-expression analyses of *SFRP1* and miRNA-1/206. Subsequently, the interaction between *SFRP1* and miRNA-1/206 was validated via dual luciferase and Western blot assays.

**Results:**

The bioinformatics analysis predicted *SFRP1* to be a target of miRNA-1/206. The expression level of the *SFRP1* was highly varied across numerous pig tissues and it was down-regulated during porcine skeletal muscle development. The expression level of the *SFRP1* was significantly higher in the embryonic skeletal compared with postnatal skeletal muscle, whereas miR-206 showed the inverse pattern of expression. A significant negative correlation was observed between the expression of miR-1/206 and *SFRP1* during porcine skeletal muscle development (p <0.05). Dual luciferase assay and Western-blot results demonstrated that *SFRP1* was a target of miR-1/206 in porcine iliac endothelial cells.

**Conclusions:**

Our results indicate that the *SFRP1* gene is regulated by miR-1/206 and potentially affects skeletal muscle development. These findings increase understanding of the biological functions and the regulation of the *SFRP1* gene in mammals.

## Background

The Wnt signaling pathway plays an essential role during embryonic and postnatal muscle development [1,2] because it regulates the expression of myogenic regulatory factors, which are essential for myogenic lineage progression and the formation of functional multinucleated myotubes [3,4]. The Wnt signaling pathway also simultaneously promotes myogenic and inhibits adipogenic differentiation within primary adult myoblasts [5]. During adult skeletal muscle regeneration, the Wnt signaling pathway is involved in satellite cell proliferation and differentiation as well as in self-renewal [6]. Secreted frizzled-related protein 1 (SFRP1) is a member of the SFRP family that inhibits Wnt signaling [7]. The SFRPs inhibit Wnt receptor binding to down-regulate pathway signaling during development [8]. The SFRP gene family has five members (*SFRP1*-*5*) in the mouse and human genomes [9], which play important roles in developmental and oncogenic processes [10,11]. The addition of recombinant SFRP1 and SFRP2 to C2C12 or primary satellite cells may inhibit myotube formation; therefore, SFRP1 and SFRP2 act to prevent myoblasts from entering the terminal differentiation process [12]. Additionally, SFRP1 also controls vascular cell proliferation in *vitro* and in *vivo* [13].

MicroRNAs are a class of small, single-stranded, noncoding RNA (~21-24 nt in length) that occur in the genomes of plants and animals. They function post-transcriptionally by interacting directly with 3′-UTRs of mRNAs to repress their expression by translational inhibition, mRNA degradation, or both [14,15]. miRNAs are involved in multiple biological processes, including development [16], cancer [17,18], cell differentiation [19], apoptosis [20], and metabolism [21]. Moreover, miRNAs play a modulatory role in the development and growth of skeletal muscles [22]. Three miRNAs, miRNA-1, −133 and −206, are specifically expressed in muscle and are considered to be myomiRs [23,24]. miRNA-1 and miRNA-133 are expressed in both cardiac and skeletal muscles [25] and miRNA-206 is only expressed in skeletal muscle [26]. miRNA-1 and miRNA-206 regulate skeletal muscle satellite cell proliferation and differentiation by repressing the paired box 7 (*Pax7*) gene [27]. These two miRNAs also promote myogenesis by targeting the histone deacetylase 4 (*HDAC4*) and the largest subunit of DNA polymerase α (*Pola1*), whereas miRNA-133 may inhibit myoblast differentiation and increase proliferation by repressing the serum response factor (*SRF*) [28,29]. Our recent study documented that miRNA-1 and miRNA-206 were abundantly and specifically expressed in porcine skeletal muscle [30].

In our previous study, we conducted mRNA and miRNA transcriptome profiling on prenatal skeletal muscle of Tongcheng pigs at 33, 65 and 90 days post-coitus (dpc) using the microarray analysis. Integrated analysis of miRNA and mRNA suggested that *SFRP1* and miRNA-1/206 exhibited opposite expression patterns and potentially interacted during prenatal skeletal muscle development. To further explore the biological functions and regulatory mechanisms of *SFRP1* gene and miRNA-1/206 in porcine muscle development, we analyzed the temporal and spatial expression patterns of miRNA-206 and *SFRP1* in prenatal and postnatal skeletal muscle at 20 developmental stages. Subsequently, the interaction between *SFRP1* and miRNA-1/206 was validated using dual luciferase and Western-blot assays.

## Methods

### Bioinformatics analysis

The public TargetScan (http://www.targetscan.org/) and PicTar (http://pictar.mdc-berlin.de/cgi-bin/PicTar_vertebrate.cgi) programs were used to predict the targets and binding sites of miRNA-1/206. A DAVID functional annotation analysis (http://david.abcc.ncifcrf.gov/) was performed to investigate the potential biological function and KEGG pathways of miRNA-1/206 targets [31]. The mRNAs and protein sequences of the *SFRP1* from different species were retrieved from the GenBank database. The isoelectric point and molecular weight of porcine *SFRP1* were predicted using the ExPASy website (http://web.expasy.org/compute_pi/). The alignment of *SFRP1*sequences and generation of the phylogenetic tree were performed by MEGA5.05 [32]. The protein localization sites in cells and the porcine *SFRP1* protein domains were predicted by the PSORT program using the K-NN method (http://psort.nibb.ac.jp/) and SMART software (http://smart.embl-heidelberg.de/), respectively.

### Animal sample collection

The Biological Studies Animal Care and Use Committee of Hubei Province, P.R. China approved the animal procedures. In this study, all animals were sacrificed at a commercial slaughterhouse according to approved procedures. Seven tissue samples, including heart, liver, spleen, lung, kidney, small intestine and *longissimus dorsi* muscle, were collected from three adult Tongcheng pigs (postnatal days 240) for the spatial expression analysis. *Longissimus dorsi* muscle samples were collected from Tongcheng pigs for dynamic expression profile analysis and were sampled at 20 developmental stages, including embryonic days 33, 40, 45, 55, 60, 70, 75, 80, 85, 90, 95, 100, and 105 (abbreviated as E33, E40, E45, E55, E60, E70, E75, E80, E85, E90, E95, E100, and E105) and postnatal days 0, 20, 40, 60, 100, 120 and 160 (abbreviated as D0, D20, D40, D60, D100, D120 and D160). At each time point, samples from three pigs were harvested as biological replicates. All samples were stored immediately in liquid nitrogen until further use.

### Isolation of RNA and reverse transcription

Total RNA was extracted according to the manufacturer’s protocol using Trizol Reagent (Invitrogen, Carlsbad, CA, USA). The total RNA concentration was determined by spectrophotometry, and sample integrity and quality were estimated by agarose gel electrophoresis and the OD_260_/OD_280_ ratio (high quality being between 1.8 and 2.0). Genomic DNA was removed using DNase I enzyme. One microgram of total RNA was reverse-transcribed into cDNA in a final volume of 20 μl using a RevertAid First Strand cDNA Synthesis Kit (MBI Fermentas, Vilnius, Lithuania) according to the manufacturer’s protocols. The cDNA was stored at −20°C.

### Real-time quantitative PCR

The expression of *SFRP1* mRNA and miRNA-206 was detected by real-time quantitative polymerase chain reaction (qPCR). The sequence of porcine miRNA-206 was obtained from the miRBase database (Accession ID:MI0013084) (http://www.mirbase.org/) [33]. Specific stem-looped primers were designed according to a previous study [34]. The gene-specific primers used for quantitative PCR are listed in Table 1. Each real-time PCR reaction was performed in a final volume of 20 μL containing 10 μL SYBR Premix Ex Taq (2×), 0.4 μL Rox Reference DyeII (50×)(TaKaRa, Dalian, China), 0.4 μL forward primer(10 μM), 0.4 μL reverse primer(10 μM), 2.0 μL template cDNA and 6.8 μL dH_2_O. PCR amplification was performed on a 7500 FAST Real-Time PCR System (Applied Biosystems, Foster City, CA, USA) under the following cycling conditions: 30 s at 95°C, followed by 40 cycles at 95°C for 5 s, 60°C for 34 s. Porcine *GAPDH* and the *U6* genes were amplified as reference controls for *SFRP1* and miRNA-206, respectively. Each reaction was performed in triplicate, and the data were analyzed by the 2^-△△^Ct method using 7500 System SDS software V 1.4.0.

**Table 1:** **Primer information**

**Gene**	**Primer sequence (5′-3′)**	**Size (bp)**
*SFRP1*-CDS	F: ACCCAGGTCTTCCTCTGCTCG	247
	R: TTGGAGGCTTCGGTGGCATT	
*SFRP1*-3′UTR	F: CTCGAGTTCTTCTAGTTCCTTCCGTAGCACC	230
	R: GCGGCCGCCGAGTGAATATTGATACATGGCAGG	
*GAPDH*	F: ATGGTGAAGGTCGGAGTGAAC	235
	R: CTCGCTCCTGGAAGATGGT	
*SFRP1* (mut-1)	F: CTCGAGTTCTTCTAGTTCCTTCCGTAGCACC	103
	R: ACAACAACACACCAATGAAATAAAACGTTTTCACAGTATT	
*SFRP1* (mut-2)	F: AATACTGTGAAAACGTTTTATTTCATTGGTGTGTTGTTGT	174
	R: GCGGCCGCCGAGTGAATATTGATACATGGCAGG	
miR-206	F: GGGTGGAATGTAAGGAA	61
	R: CTCAACTGGTGTCGTGGAGTC	
*U6*	F:GCTTCGGCAGCACATATACTAAAAT	89
	R:CGCTTCACGAATTTGCGTGTCAT	
miR-206 -RT	CTCAACTGGTGTCGTGGAGTCGGCAATTCAGTTGAGTCACACAC	
*U6*-RT	CGCTTCACGAATTTGCGTGTCAT	

### Plasmid construct

A 230 bp fragment encompassing a partial *SFRP1* 3′-UTR containing miRNA-1/206 binding sites was cloned from a Tongcheng pig using gene-specific primers (Table 1). This fragment was inserted downstream of the *Renilla* luciferase open reading frame in the psiCheck-2 vector (Promega, USA) using NotI and XhoI restriction sites. The mutant *SFRP1* 3′-UTR sequence, which had a 7 bp deletion in the binding site, was cloned by bridge PCR and inserted into the final destination vectors to construct the mutated vector. To construct the *SFRP1* over-expression vector, a 247 bp fragment containing the coding sequence of *SFRP1* was cloned into the NheI and XhoI restriction sites of the psiCHECK-2 vector to replace the *Renilla* coding sequence, resulting in *SFRP1*-CDS-3′-UTR-psiCHECK-2. All the PCR products were confirmed by direct sequencing.

### Cell culture and dual luciferase reporter assay

Porcine iliac endothelial cells (PIECs; obtained from the Institute of Biochemistry and Cell Biology, Chinese Academy of Sciences, P.R. China) were cultured in Dulbecco’s modified Eagle's medium with high glucose (Gibco, Invitrogen, Carlsbad, CA, USA), supplemented with 10% fetal bovine serum (Gibco), 1% glutamine, and 1% penicillin/streptomycin (Gibco). The cells were incubated at 37°C in 5% CO_2_. Chemically synthesized miRNA-1/206 or the negative control duplexes (Gene Pharma, Shanghai, China) were transfected into the PIECs in combination with a luciferase reporter containing wild-type or mutant *SFRP1* 3′UTR using Lipofectamine 2000 reagent(Gibco) in 24-well plates. Each transfection was performed in triplicate. At 48 h after transfection, all the cells were harvested. *Renilla* and Firefly luciferase activities were measured with the Dual Luciferase Assay System (Promega, Madison, WI, USA) in a TD-20/20 luminometer (Turner Biosystems, Sunnyvale, CA, USA). The *Renilla* luciferase signal was normalized to the Firefly luciferase signal. The normalized *Renilla* luciferase activity was compared with the control, miRNA-1/206 and the mutant groups using Student’s *t*-test (p < 0.05) with SPSS 15.0 software.

### Western-blot analysis

PIECs, transfected with the indicated plasmids, were harvested at 48 h after transfection, and washed twice in phosphate-buffered saline. The total protein was extracted using M-PER Mammalian Protein Extraction Reagent (Thermo Scientific, USA). Protein concentrations were measured with a Pierce BCA Protein Assay Kit (Thermo Scientific, USA) according to the manufacturer’s instructions. Western-blot analysis was performed as follows: 20 μg of protein per lane was resolved by sodium dodecyl sulfate polyacrylamide gel electrophoresis and transferred to polyvinylidene difluoride (PVDF) membranes (Millipore). The PVDF membranes were probed with primary antibodies (Santa Cruz Biotech., Santa Cruz, CA, USA) at a 1:1,000 dilution and with β-actin (Santa Cruz Biotech., Santa Cruz, CA, USA) at a 1:5,000 dilution as a control. The membranes were further incubated with an horseradish-peroxidase-conjugated secondary antibody (Zymed, San Diego, CA, USA) at a 1:10,000 dilution.

## Results

### Target prediction of miRNA-1/206 and bioinformatic analysis

**Figure 1:**
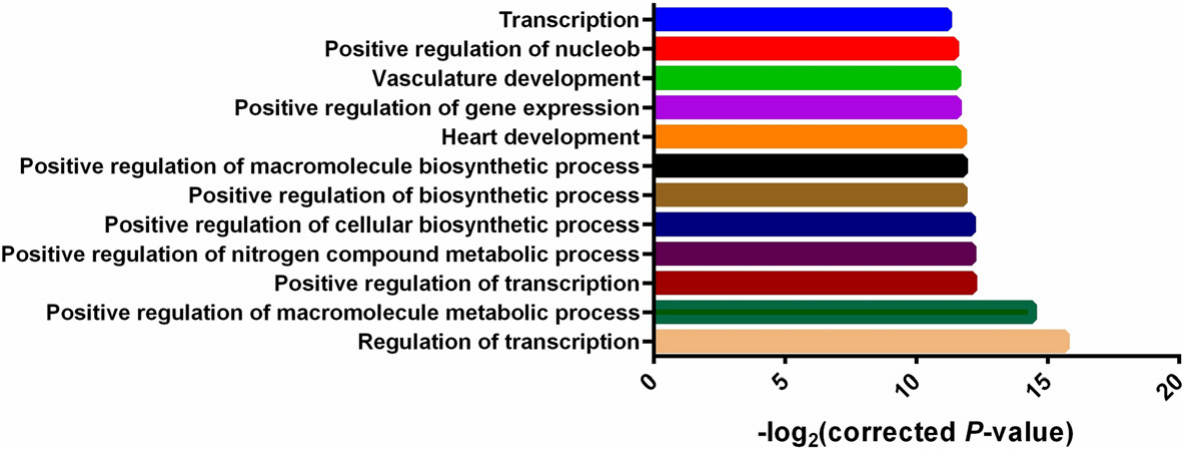
**Top 10 GO biological process terms significantly enriched in for target genes of miRNA-1/206.** GO analysis was conducted with a DAVID functional annotation program.

The candidate targets of miRNA-1/206 were predicted using TargetScan and PicTar programs. 258 candidate targets were predicted by both programs. To explore the biological function of the candidate genes, a DAVID functional annotation analysis was performed using the thresholds EASE adjusted to p < 0.05. The Gene Ontology (GO) analysis revealed that these targets were significantly enriched in the regulation of transcription, positive regulation of macromolecule metabolic processes, positive regulation of nitrogen compound metabolic processes, positive regulation of cellular biosynthetic processes and other biological processes (Figure 1). KEGG pathway analysis showed that these targets were significantly enriched in four pathways (p <0.05), which were SNARE interactions in vesicular transport, the Wnt signaling pathway, small cell lung cancer and the Neurotrophin signaling pathway (Table 2). Seven putative targets, including *CCND1*, *SFRP1*, *CCND2*, *PPP2R5A*, *NFAT5*, *DAAM1* and *FZD7*, participated in the Wnt signaling pathway. Therefore, the *SFRP1* gene, which was predicted to be a target of miRNA-1/206 and was involved in the Wnt signaling pathway, was selected for further study.

**Table 2:** **Significantly enriched KEGG pathways of target genes**

**Category**	**Term**	**Count**	**%**	**p-value**	**Genes**
KEGG_PATHWAY	hsa04130:SNARE interactions in vesicular transport	4	1.55	0.023	*BET1, VAMP4, VAMP2, SNAP25*
KEGG_PATHWAY	hsa04310:Wnt signaling pathway	7	2.71	0.034	*CCND1,* ***SFRP1*** *, CCND2, FZD7, PPP2R5A, NFAT5, DAAM1,*
KEGG_PATHWAY	hsa05222:Small cell lung cancer	5	1.94	0.046	*CCND1, PIAS3, CDK6, RARB, FN1*
KEGG_PATHWAY	hsa04722:Neurotrophin signaling pathway	6	2.23	0.049	*BDNF, YWHAZ, RAP1A, RAP1B, NGFR, CALM2*

*SFRP1* is involved in the Wnt signaling pathway and selected for further study.

### Sequence analysis of the porcine *SFRP1* gene

**Figure 2:**
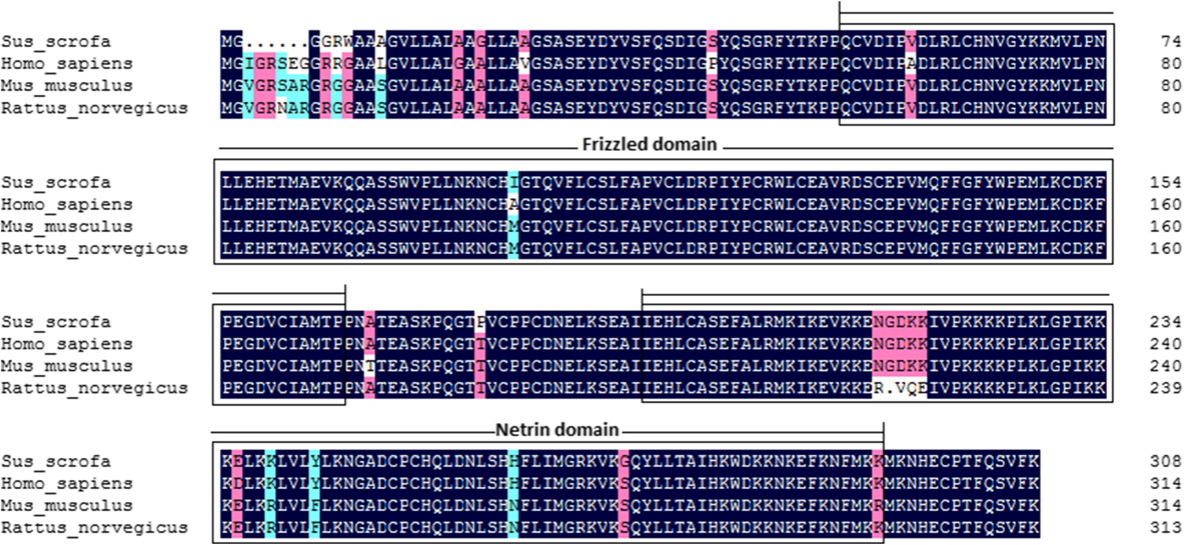
**Comparison of porcine** ***SFRP1*** **sequence (GenBank accession ID: XP_003359916.2) with those of human (GenBank accession ID: NP_003003.3), mouse (GenBank accession ID: NP_038862.2) and rat (GenBank accession no. NP_001263641.1) sequence.** Shading shows identical and similar amino acid residues among the four species. Common structural domains are indicated by boxes including a Frizzled domain and a Netrin C-terminal domain.

**Figure 3:**
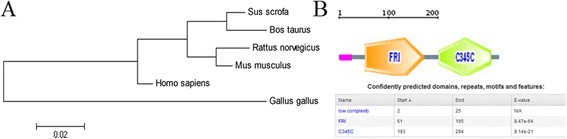
**Phylogenetic tree and domains of porcine** ***SFRP1*** **protein. (A)** Phylogenetic tree of *SFRP1* from different species. The GenBank accession numbers of those sequences are as follows: pig, XP_003359916.2; human, NP_003003.3; cattle,NP_776885.1; rat, NP_001263641.1; mouse, NP_038862.2, and chicken, NP_989884.1. **(B)** Porcine *SFRP1* protein domains. The porcine *SFRP1* precursor protein contains a Frizzled domain and a Netrin C-terminal domain.

The *SFRP1* protein sequences (Accession ID: XM_003359868.3) were retrieved from the GenBank database. First, the amino acid sequence of porcine SFRP1 was compared with those of the human, mouse and rat, and the alignment results showed that the protein sequence of the pig had a six amino acid depletion. The porcine *SFRP1* polypeptide exhibited 96.09%, 94.26% and 93.31% similarity with the human, mouse and rat homologs, respectively (Figure 2). Based on the phylogenetic tree analysis, the protein sequence of the pig was closely related to that of cow, while the rat and mouse formed another closely related group, and these four species formed a group with the humans (Figure 3A). SMART software was used to predict *SFRP1* protein domains. The precursor protein of *SFRP1* contained a Frizzled domain and a Netrin C-terminal domain(Figure 3B). The sites of *SFRP1* protein localization had a 44.4% possibility of being extracellular, a 22.2% possibility of being cytoplasmic and a 22.2% possibility of being in the endoplasmic reticulum. Additionally, the theoretical isoelectric point (pI) and molecular weight (Mw) of *SFRP1* were 9.05 and 34.79 KDa, respectively.

### Distribution of porcine *SFRP1* mRNA and miRNA-206 in tissues

**Figure 4:**
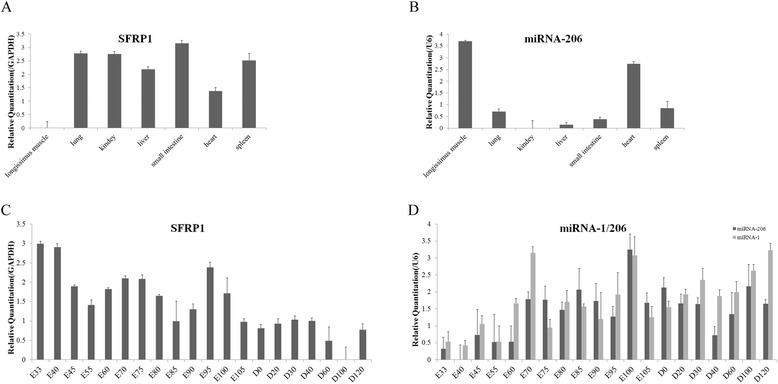
**Relative expression of** ***SFRP1*** **and miRNA-206 in the different tissues and in skeletal muscle during development of Tongcheng pigs. (A)** Tissue distribution of *SFRP1* in the adult Tongcheng pig. **(B)** Tissue distribution of miRNA-206 in the adult Tongcheng pig. **(C)** Relative expression of *SFRP1* mRNA and **(D)** miRNA-1/206 in porcine skeletal muscle from Tongcheng pigs at different developmental stages (20 stages). miRNA-1 expression in Tongcheng pigs is cited from Tang et al. [35]. The values are the average (±SE) levels of *SFRP1* mRNA and miRNA-1/206 from three independent experiments normalized to *GAPDH* and *U6*, respectively. In each group, the lowest expression value was arbitrarily set to 0 by log_10_ transformation to evaluate the relative expression levels.

The expression of *SFRP1* and miRNA-206 was measured in seven tissues from adult Tongcheng pigs. The *SFRP1* gene was highly expressed in the kidney, liver, lung, spleen and small intestine, moderately expressed in the heart, and weakly expressed in the *longissimus dorsi* muscle (Figure 4A). However, miRNA-206 was abundantly expressed in the *longissimus dorsi* muscle and heart, and was weakly expressed in the other tissues (Figure 4B).

### Developmental expression of porcine *SFRP1* mRNA and miRNA-206

We collected the *longissimus dorsi* muscle from 20 prenatal and postnatal developmental stages of Tongcheng pigs. Quantitative real-time PCR indicated that *SFRP1* was highly expressed at the E33 stage, and was then down-regulated from E33 to E55. It was then up-regulated from E55 to E70 and down-regulated from E70 to E85. Subsequently, it was up-regulated from the E85 to E95, and then down-regulated again from E95 to D0. The expression of *SFRP1* was maintained at a stable low level in postnatal skeletal muscle. In postnatal myogenesis, *SFRP1* was consistently expressed from days 0 to 40. Subsequently, it decreased and reached a minimum at D100 (Figure 4C). The expression of miRNA-206 was weak in skeletal muscle in the early embryonic stages, but then remained stable with high levels of expression in the remaining prenatal stages, although some fluctuations occurred. The expression level of miRNA-206 reached a peak at E100. In postnatal muscle, miRNA-206 was down-regulated from D0 to D40 and was up-regulated from D40 to D100 (Figure 4D).

### Co-expression analysis of *SFRP1* and microRNA-1/206

**Figure 5:**
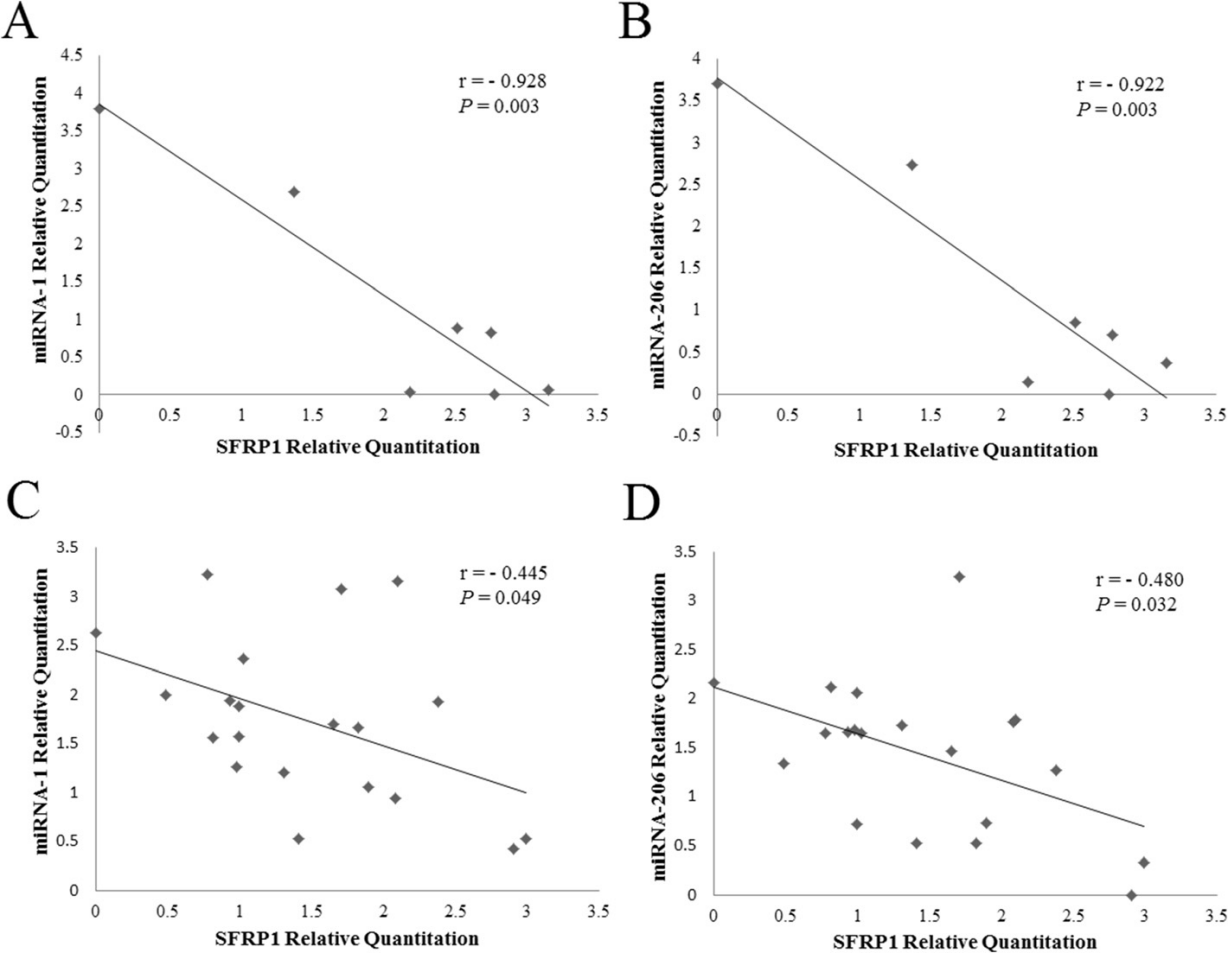
**Correlation analyses of miRNA-1/206 and** ***SFRP1*** **expression. (A)** Pearson’s correlation between *SFRP1* and miRNA-1 expression in different tissues. **(B)** Pearson’s correlation between *SFRP1* and miRNA-206 expression in different tissues. **(C)** Pearson’s correlation between *SFRP1* and miRNA-1 expression during skeletal muscle development. **(D)** Pearson’s correlation between *SFRP1* and miRNA-206 expression during skeletal muscle development.

Tang et al. have explored the spatial and dynamic expression of miRNA-1 in the Tongcheng pig (Figure 4D) [35]. According to their study and this study, miRNA-1 and miRNA-206 were abundantly expressed in skeletal muscle and weakly expressed in other tissues. In contrast, *SFRP1* was expressed at very low levels in adult skeletal muscle. The co-expression analysis revealed that the *SFPR1* mRNA was significantly negatively correlated with miRNA-1 (Pearson’s R_*SFRP1*/miRNA-1_ = −0.928, p _value_ = 0.003) (Figure 5A) and miRNA-206 (Pearson’s R_*SFRP1*/miRNA-1_ = −0.922, p _value_ = 0.003) (Figure 5B) at the mRNA level in different tissues adult tissues.

During skeletal muscle development, *SFPR1* had a higher expression level in the prenatal stages than in the postnatal stages. However, in contrast to *SFRP1*, miRNA-1/206 exhibited a relatively higher level of expression in postnatal muscle compared with prenatal muscle. *SFRP1* mRNA was significantly negatively correlated with miRNA-1/206 [Pearson’s R_*SFRP1*/miRNA-1_ = −0.445, p_value_ = 0.032 (Figure 5C); Pearson’s R_*SFRP1*/miRNA-206_ = −0.480, p _value_ = 0.049(Figure 5D)]. These results indicated that *SFRP1* expression might be regulated by miR-1/206 in pigs.

### *SFRP1* is a putative target of microRNA-1/206

**Figure 6:**
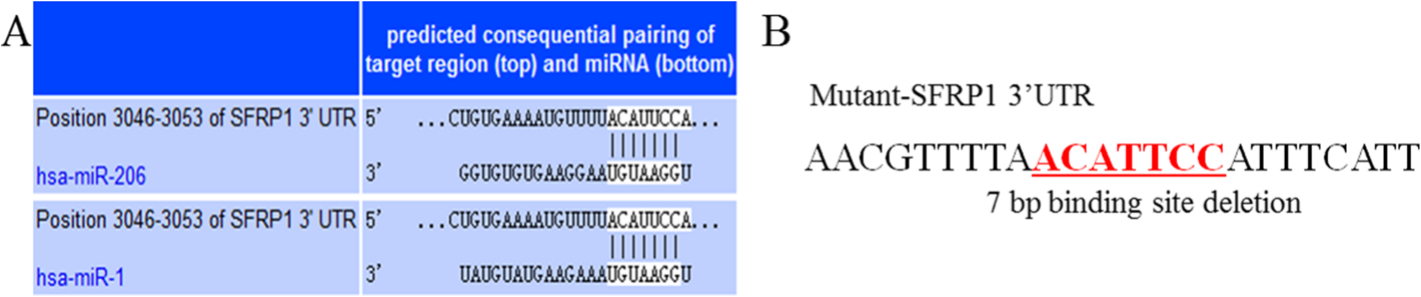
***SFRP1*** **3′-UTR has miR-1/206 target binding sites. (A)** *SFRP1* was predicted as a target of microRNA-1/206. **(B)** Schematic of the predicted miRNA-1 and miRNA-206 binding sites (underlined) in the 3′ UTR of *SFRP1*. The binding site region was deleted in the mutant 3′ -UTR reporters.

**Figure 7:**
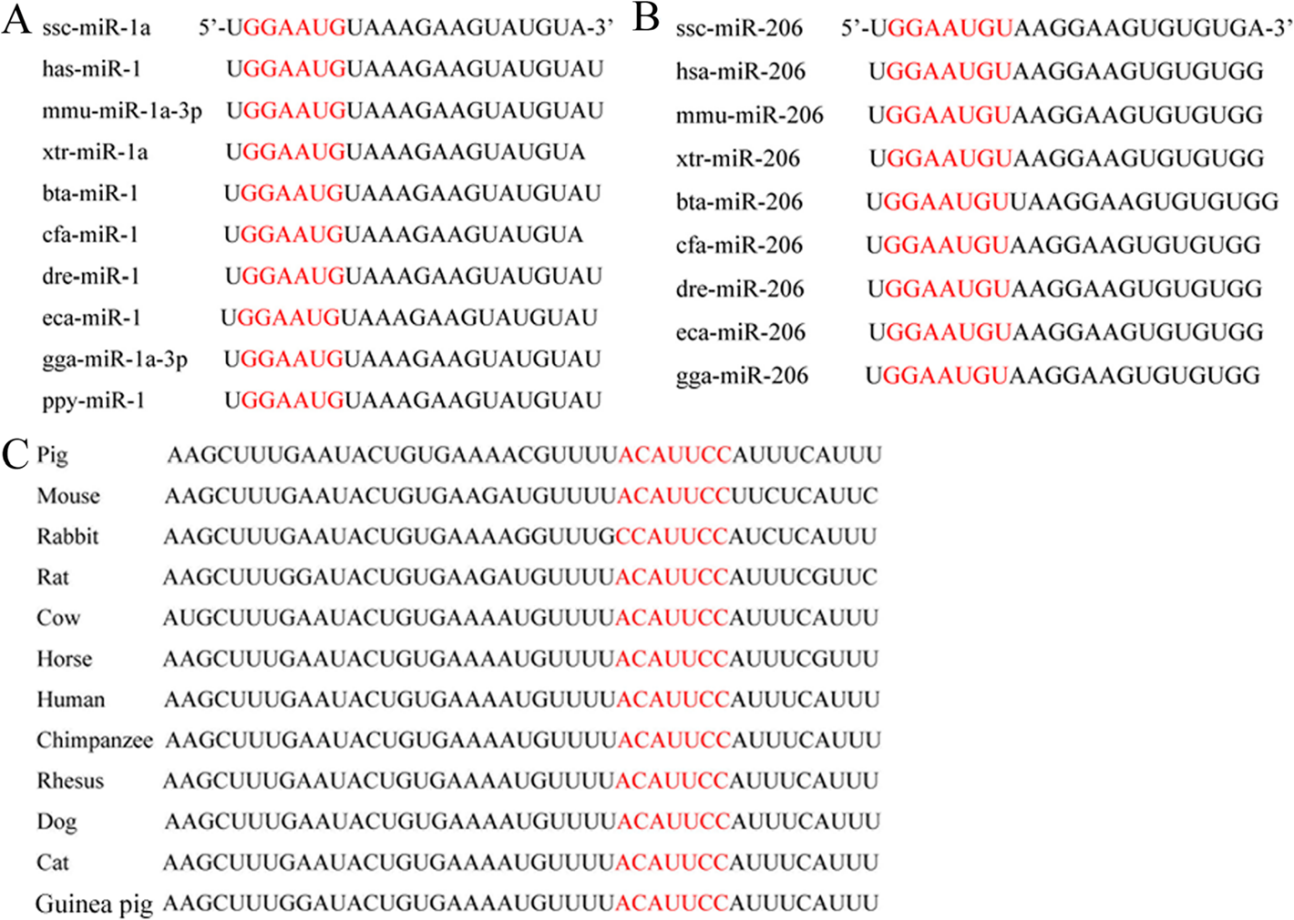
**Predicted miRNA-1 and miRNA-206 binding sites (highlighted in red) in the 3′-UTR of** ***SFRP1*** **showing species conservation. (A)** Seed sequence of miRNA-1 showing species conservation. **(B)** Seed sequence of miRNA-206 showing species conservation. **(C)** Binding sites (highlighted in red) in the 3′-UTR of *SFRP1* showing species conservation.

The prediction from the miRNA-mRNA profiles and bioinformatics suggested that *SFRP1* was potentially a miRNA-1/206 target in pigs. In the 3′-UTR region of *SFRP1* mRNA, a putative binding site was identified (7 bp conserved homology) (Figure 6A). The seed sequence of miRNA-1/206 and the target-binding site between *SFRP1* and miRNA-1/ 206 were highly conserved across mammals (Figure 7).

**Figure 8:**
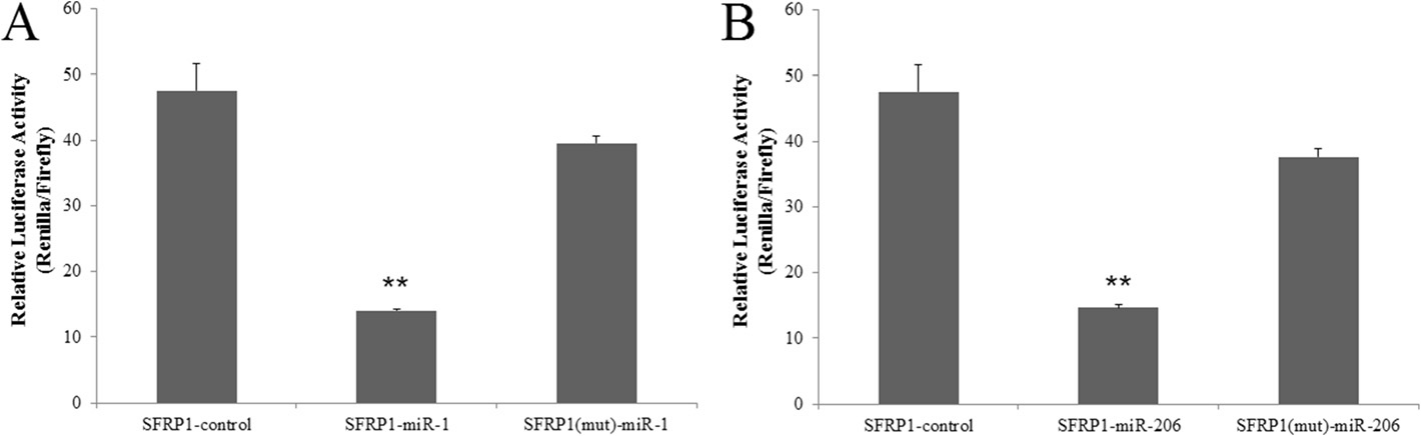
**Validating SFRP1 as a positive target for miRNA-1 and miRNA-206.** Cotransfection of porcine pre-miRNA-1 **(A)** and pre-miRNA-206 **(B)** or control and porcine SFRP1 UTR-derived psiCHECK-2 construct or mutant in PIEC cells. Renilla activity at 48 h post-transfection shows a significant decrease in normalized values compared with the control and mutant. Three replicates were performed for each group. **Indicates a p-value of less than 0.01 in Student’s t-test.

To validate whether *SFRP1* was directly targeted by miRNA-1/206 in pigs, we constructed the psiCheck2-*SFRP1*-3′-UTR , a luciferase reporter vector. Subsequently, miRNA-1and miRNA-206 mimics and a normal control (NC) were co-transfected into PIECs, and luciferase activity was detected. The miRNA-1 mimic-transfected group exhibited 68.29% less luciferase activity compared with the NC group (p < 0.01) and the miRNA-206 mimic-transfected group exhibited 71.25% less luciferase activity compared with the control (p < 0.01) (Figure 8). To further validate the specific target site, the binding region of the *SFRP1* 3′-UTR was mutated by bridge PCR (Figure 6B). The luciferase activity of the psiCHECK-2-*SFRP1*–3′-UTR (mut) was not significantly decreased by both the miRNA-1 and miRNA-206 mimics (12.39% of control for the miRNA-1 mimic-transfected group and 16.76% of control for the miRNA-206 mimic-transfected group) (p >0.05) (Figure 8).

**Figure 9:**
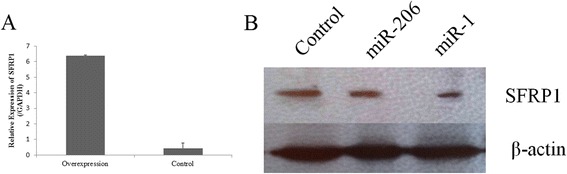
**The miRNA-1 and miRNA-206 regulate** ***SFRP1*** **at the protein level. (A)** Overexpression of porcine *SFRP1* in PIEC cells. Histogram indicates overexpression of *SFRP1* 48 h after transfection. **(B)** miRNA-1 and miRNA-206 down-regulated the *SFRP1* at the protein level. The expression of *SFRP1* was normalized against β-actin.

Then, we determined whether *SFRP1* was affected by miRNA-1/206 at the protein level. The overexpression vector of *SFRP1* was constructed and transfected into PIECs, and the quantitative real-time PCR (qPCR) results showed that the expression of *SFRP1* was increased approximately 16-fold compared with the NC group. This result indicated that the *SFRP1* overexpression vector was successful (Figure 9A). The porcine *SFRP1*-CDS-3′-UTR-psiCHECK-2 vector was constructed, and it was co-transfected with miRNA-1 and miRNA-206 mimics in PIECs. The Western blot results showed that the protein level of *SFRP1* in the groups containing miRNA-1/206 mimics was decreased compared with the NC group (Figure 9B). These results suggested that the *SFRP1* gene was a target of miRNA-1/206.

## Discussion

We predicted the targets of miRNA-1/206 with the TargetScan and PicTar programs. Among the putative targets, many genes had been validated by previous studies, such as *HDAC4* [29], *GJA1*, *KCNJ2* [36], *Pola1* [28], and *Met* [37]. However, all these results were based on myoblast C2C12 cells in vitro. Few reports have considered miRNA-1/206 targets during skeletal muscle development in *vivo*, particularly in pigs. To discover potential targets of miRNA-1/206 during swine myogenesis, we conducted GO and KEGG pathway analyses of targets based on the prediction data. The results suggested that these targets were significantly enriched in the Wnt signaling pathway (p < 0.05). Wnts signaling proteins are secreted proteins that function in differentiation, embryonic development and cell proliferation [38]. The Wnt pathways play an important role in the formation of muscle fibers during prenatal [39] and postnatal myogenesis with the activation of stem cells in the adult muscles [40,41]. *SFRP1*, a secreted antagonist of the Wnt-Frizzled pathway, was predicted to be a target of miRNA-1/206 and to participate in the Wnt signaling pathway. The expression of *SFRP1* mRNA was up-regulated in muscle regeneration [42] and in skeletal muscle after denervation [43]. Additionally, an impairment of the Wnt-Frizzled pathway via *SFRP1* over-expression controlled proliferation and neovascularization after muscle ischemia [13]. A comparison of the amino acid sequence of pig *SFRP1* with those of human, rat and mouse demonstrated remarkably high similarity across species, and the frizzled domain and netrin C-terminal domain were especially highly conserved.

Previous studies reported *SFRP1* expression at the mRNA level in various tissues, such as the brain, kidney and heart, and that the transcript was present both in the adult and during embryogenesis [7,44]. Moreover, *SFRP1* was weakly expressed in matured skeletal muscles [45]. The qPCR results for *SFRP1* in various adult porcine tissues were mostly consistent with these reports. These results indicated that *SFRP1* had almost no antagonistic effect in adult skeletal muscle. Postnatal muscle growth is largely determined by the total number of fibers, which is determined by two major waves of fiber generation before birth: primary muscle fiber formation at 35–60 dpc and assemblage of secondary muscle fibers at 54–90 dpc [46-48]. The *SFRP1* gene was down-regulated in skeletal muscle from E33 to E55 in Tongcheng pigs, indicating that *SFRP1* was primarily involved in the formation of primary muscle fibers. We also found that the expression of *SFRP1* was higher in embryonic skeletal muscle compared with postnatal skeletal muscle. miRNA-206, meanwhile, was abundantly expressed in skeletal muscle and heart, and up-regulated from pre- to postnatal-stage skeletal muscle. These results demonstrated that miR-206 plays a key role in skeletal muscle development in pigs. *SFRP1* primarily affected skeletal muscle development in embryonic stages. Wnts signaling contributes to the overall process of myogenesis by activating myogenic regulatory factor genes such as Myf5 and MyoD [49]. miRNA-1/206 promoted skeletal muscle satellite cell proliferation and differentiation [27] and *SFRP1* might inhibit myoblast differentiation [12]. The temporal expression patterns of miRNA-1/206 and *SFRP1* in Tongcheng pigs were consistent with these previous findings.

The mRNA-miRNA co-expression correlation analysis was reported to identify the putative targets of miRNA [50,51], and this method could improve the positive rate for identifying the mRNA target genes of miRNA. miRNA-1 and miRNA-206 were abundant in the postnatal stages and were at low levels in the prenatal stages of muscle development, while *SFRP1* exhibited an opposite expression patterns. Correlation analysis revealed that the SFPR1 was significantly negatively correlated with miRNA-1/206 (p < 0.05), and these results indicate that *SFRP1* is potentially regulated by miRNA-1/206.

The interaction between *SFRP1* and miRNA-1/206 in pigs has not been previously reported. This study demonstrated that *SFRP1* expression was regulated by miRNA-1/206. The *SFRP1* 3′-UTR sequence around the miRNA-1/206 target sites and the seed sequence of mature miRNA-1/206 are well conserved in mammals, which suggests that the target region is important in *SFRP1* regulation and that the regulation of *SFRP1* by miRNA-1/206 may also exist in other species. Similar results were observed in other miRNA studies [30,52].

The luciferase activity of psiCHECK-2 containing the *SFRP1* 3′-UTR sequence was significantly decreased by co-transfection with miRNA-1/206 mimics (p < 0.05). However, with the *SFRP1* 3′-UTR mutant sequence, activity was not significantly decreased (p > 0.05). These results indicated that the target binding site was specific and unique in pigs, and that miRNA-1/206 might repress *SFRP1* expression by degrading the mRNA transcripts. We propose that *SFRP1* is regulated by miRNA-1/206, is >involved in the proliferation of muscle cells, and affects prenatal skeletal muscle development. Moreover, we explored the interactions between *SFRP1* and miRNA-1/206 at the protein level, and Western-blot analysis confirmed that *SFRP1* was significantly down-regulated by miRNA-1/206.

## Conclusions

In summary, we predicted the target genes of miRNA-1/206 and performed a functional annotation of the target genes. We performed a molecular characterization analysis of the porcine *SFRP1* gene,, which was one of the predicted targets for miRNA-1/206. We also explored the spatial-temporal profile of *SFRP1* mRNA and miRNA-206 in adult tissues and in skeletal muscle tissues during development in Tongcheng pigs. The results indicated that *SFRP1* was primarily involved in prenatal skeletal muscle development. Finally, we verified that porcine *SFRP1* was a target of miRNA-1/206 using dual luciferase and Western blot assay. These results increase our understanding of the biological functions of *SFRP1* and miRNA-1/206 in skeletal muscle development.

## Abbreviations

*SFRP1*: Secreted frizzled-related protein 1
*MRFs*: Myogenic regulatory factors
PIECs: Porcine iliac endothelial cells
*HDAC4*: Histone deacetylase 4
*Pola1*: the largest subunit of DNA polymerase α
miRNA: microRNA
NC: Normal control
GAPDH: Glyceraldehyde-3-phosphate dehydrogenase
qPCR: quantitative real-time PCR
